# Graphene@Curcumin-Copper Paintable Coatings for the Prevention of Nosocomial Microbial Infection

**DOI:** 10.3390/molecules28062814

**Published:** 2023-03-20

**Authors:** Mohammad Oves, Mohammad Omaish Ansari, Mohammad Shahnawaze Ansari, Adnan Memić

**Affiliations:** 1 Center of Excellence in Environmental Studies, King Abdulaziz University, Jeddah 21589, Saudi Arabia; 2 Center of Nanotechnology, King Abdulaziz University, Jeddah 21589, Saudi Arabia

**Keywords:** antimicrobial, coating, graphene, curcumin, copper, *Pseudomonas aeruginosa*, *E. coli*

## Abstract

The rise of antimicrobial resistance has brought into focus the urgent need for the next generation of antimicrobial coating. Specifically, the coating of suitable antimicrobial nanomaterials on contact surfaces seems to be an effective method for the disinfection/contact killing of microorganisms. In this study, the antimicrobial coatings of graphene@curcumin-copper (GN@CR-Cu) were prepared using a chemical synthesis methodology. Thus, the prepared GN@CR-Cu slurry was successfully coated on different contact surfaces, and subsequently, the GO in the composite was reduced to graphene (GN) by low-temperature heating/sunlight exposure. Scanning electron microscopy was used to characterize the coated GN@CR-Cu for the coating properties, X-ray photon scattering were used for structural characterization and material confirmation. From the morphological analysis, it was seen that CR and Cu were uniformly distributed throughout the GN network. The nanocomposite coating showed antimicrobial properties by contact-killing mechanisms, which was confirmed by zone inhibition and scanning electron microscopy. The materials showed maximum antibacterial activity against *E. coli* (24 ± 0.50 mm) followed by *P. aeruginosa* (18 ± 0.25 mm) at 25 µg/mL spot inoculation on the solid media plate, and a similar trend was observed in the minimum inhibition concentration (80 µg/mL) and bactericidal concentration (160 µg/mL) in liquid media. The synthesized materials showed excellent activity against *E. coli* and *P. aeruginosa*. These materials, when coated on different contact surfaces such medical devices, might significantly reduce the risk of nosocomial infection.

## 1. Introduction

Infectious diseases that are caused by microorganisms can lead to serious health complications, including death, for both humans and animals. The attachment of microbes to surfaces leading to the formation of biofilms poses a particularly serious threat. Many industries, ranging from healthcare systems, the food and water industry, as well as the oil and gas industry, suffer huge losses due to complications as a result of biofilm formation [1]. Further challenges arise when considering that microbes adhering to the surfaces can be transferred when touched, thereby spreading microbial colonization. During the global COVID-19 outbreak, the development of antimicrobial surfaces attracted the attention of scientists worldwide. The development of antimicrobial coating could be an effective strategy to prevent microbial spread in general and specifically biofilm formation. [2]. To achieve these goals, several approaches have been proposed [3]. For example, recently, a combination of different nanomaterials, such as graphene (GN), metals and metal oxides, or natural antibacterial materials, have been proposed as antimicrobial coatings. Such a combination of materials could have exciting synergistic or additional effects when compared to the individual components developed for anti-infective surface deployment.

Some recently designed GN-based nanomaterials are known to exhibit antimicrobial activity towards bacteria [4], fungi [5], and viruses [6]. The direct physicochemical interaction between the GN-based materials and microbes can lead to the damage of the cellular components, principally the proteins, lipids, and nucleic acids [7]. The high affinity of GN-based materials towards membrane proteoglycans leads to membrane damage. Another mechanism of action is the further internal leakage of GN-based materials into cells which can lead to the inhibition of DNA/RNA replication [8]. Therefore, it is proposed that the mode of action of GN towards bacteria is by physical means as well as by chemical means [9]. The physical means operate by the direct contact of microorganisms with the sharp edges of GN. For example, Akhavan et al. [10] showed that the GN-based materials could potentially encapsulate microorganisms for their isolation but also act as an effective photothermal agent for the inactivation of the GN-wrapped microorganisms. Pham et al. [11] similarly showed that the density of GN edges significantly affects its antibacterial activity. The researchers proposed that GN sheets act as blades cutting through the cell membrane and induce pores, leading to their rupture leading to bacterial death. On the other hand, chemical mechanisms involve oxidative stress and the generation of reactive oxygen species (ROS) responsible for the inactivation of microorganisms [12].

Among metallic particles, many research groups have shown that copper (Cu) and its oxides have antimicrobial properties [13,14,15,16,17,18,19,20]. The mechanism of killing involves bacterial cell wall damage leading to the loss of cytoplasmic content [21]. Apart from this, the reactive oxygen species can induce even greater damage to organelles and even lead to nucleic acid degradation [22]. Cu has also been found to possess high antiviral properties as well [23]. Bleichert et al. [24] showed that attenuated vaccine strain vaccinia virus and virulent MPXV Copenhagen were inhibited within 3 min of exposure to Cu. Similarly, Noyce et al. [25] showed that the Cu alloys (61–95% Cu) effectively killed *E. coli* at room temperature. They also found that samples with a high percentage of Cu possessed the highest antibacterial properties. Similar results were also reported by Wilks et al. [26], who showed that a Cu content of 85% or more showed good antibacterial activities. Similar effects of Cu were found against the vegetative and spore forms of *Clostridium difficile* and a significant reduction in survival of the *C. difficile* vegetative cells and spores was observed after 24–48 h [27]. From the above discussion, it appears that the amount of Cu can have a direct effect on the contact killing of microorganisms, including both bacteria and viruses.

Among naturally occurring antimicrobial materials, curcumin (CR) has been widely used since its antibacterial properties were demonstrated by Schraufstatter and Bernt in 1949 [28]. CR promotes recombinant protein overexpression, thereby leading to an apoptosis-like response in bacteria [29]. Several investigations revealed that CR had antibacterial effects on both Gram-positive and Gram-negative bacteria [30]. CR antibacterial activity includes bacterial membrane rupture, the suppression of virulence factor synthesis and suppression of biofilm formation, and the activation of oxidative stress [31,32]. Recently, Oves et al. [33] has investigated CR- and ZnO-glazed GN for the successful growth inhibition of a Methicillin-resistant bacterial strain of *Staphylococcus aureus*.

**Figure 1:**
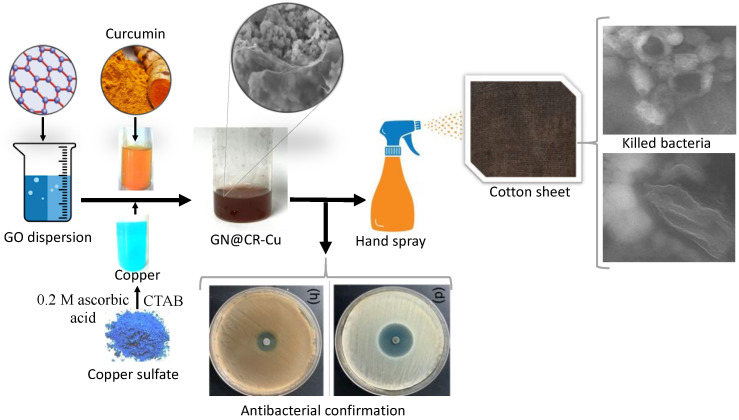
Schematic representation of the synthesis and antibacterial application of GN@CR-Cu.

Due to the antibacterial properties of both GN and Cu paired with CR, it can be interpreted that their combination will be highly effective in combating different types of bacteria via contact killing. Thus, in this work, solutions of Cu particles and CR dispersed in graphene oxide (GO) gel was prepared. The prepared dispersion was applied on contact surfaces and its subsequent heat treatment resulted in the reduction of GO into reduced-graphene oxide (rGO) (Figure 1). The Cu, CR, and reduced-graphene oxide coatings were thereafter tested for the contact killing of *Pseudomonas aeruginosa* (*P. aeruginosa*) and *Escherichia coli* (*E. coli*).

## 2. Results and Discussion

### 2.1. Morphological Analysis

**Figure 2:**
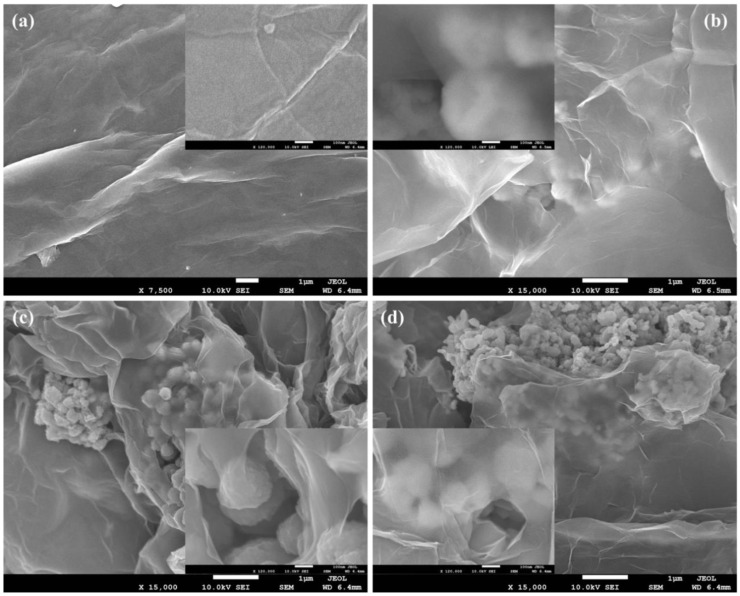
Low and high magnification (insets) FESEM images of (**a**) GN; (**b**) GN@Cu; (**c**) GN@CR; and (**d**) GN@CR-Cu.

FESEM images of GN, GN@Cu, GN@CR, and GN@CR-Cu at low and high magnifications (insets) are shown in Figure 2a–d. The folded sheet-type structure can be very clearly observed in Figure 2a, which is a known feature of GN. When it comes to GN@Cu (Figure 2b), solid polyhedral-shaped Cu particles are visible below and above the GN sheets. It is evident from the inset of Figure 2a that the Cu particles are a bit blurry because of their presence underneath a very thin and nearly transparent layer of GN. On the other hand, the well-dispersed fluffy circular geometries of the CR particles with GN sheets can be observed in Figure 2c. The CR particles are easily detectable in a small void between the single and multi-layered GN sheets in the inset of Figure 2c. As far as the GN@CR-Cu composite is concerned (Figure 2d), the sample is homogeneous with Cu, and CR particles are uniformly distributed. The inset of Figure 2d also reveals that the polyhedral solid geometries of the Cu particles and slightly circular-shaped CR particles are sandwiched between the ultrafine layers of GN. The FESEM image of CR and Cu nanoparticles can be seen in Figure S1.

**Figure 3:**
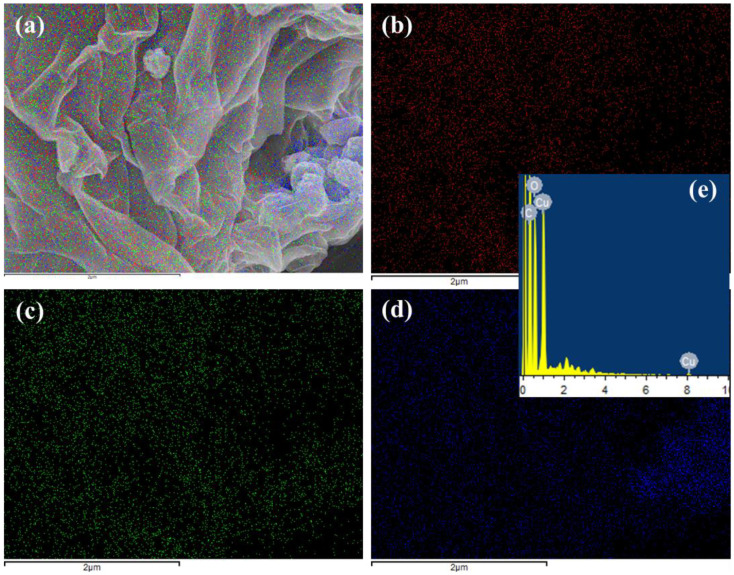
Elemental mapping images of (**a**) GN@CR-Cu; (**b**) C; (**c**) O; (**d**) Cu; and (**e**) EDS spectrum of GN@CR-Cu.

Figure 3a–e shows the elemental mapping and quantitative analysis of the as-synthesized GN@CR-Cu sample recorded by using EDS. The mixed electron image of the GN@CR-Cu (Figure 3a), C in red (Figure 3b), O in green (Figure 3c), and Cu in blue shows that the prepared sample, in the form of a composite, has all three (GN, Cu, and CR) components with uniform distribution. Figure 3e illustrates the EDS spectrum consisting of C, O, and Cu peaks, indicating that the GN@CR-Cu composite was successfully fabricated.

**Figure 4:**
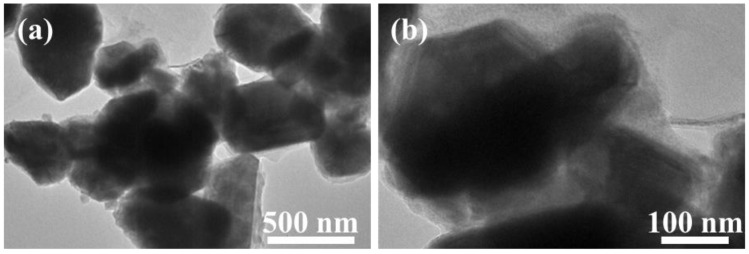
(**a**) Low magnification; (**b**) High magnification HRTEM images of GN@CR-Cu.

Figure 4a,b shows low and high magnification HRTEM images of GN@CR-Cu. Figure 4a reveals that the sample is well-formed and homogeneous with a uniform amalgamation of GN, Cu, and CR. All three components, the sheet-like layered structure of GN, the almost hexagonal/polyhedral-shaped solid surface of Cu, and the fluffy/circular geometry of CR particles can be easily identified in the image Figure 4b.

### 2.2. X-ray Photoelectron Spectroscopy (XPS)

**Figure 5:**
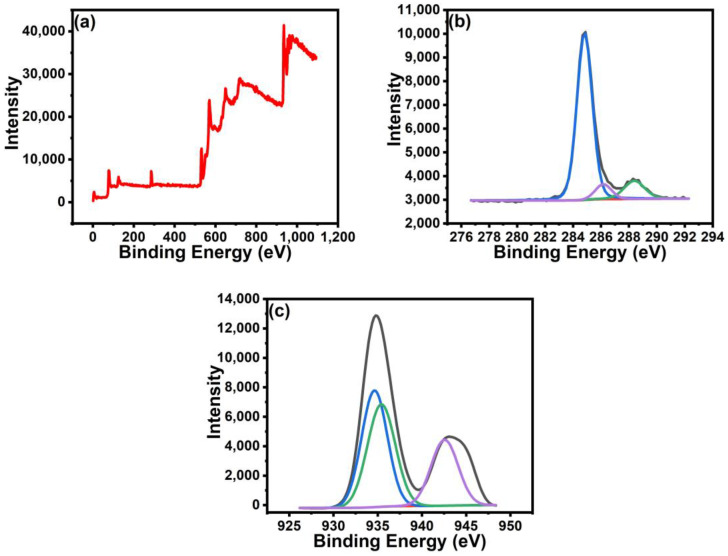
XPS spectra of GN@CR-Cu composite: (**a**) survey scan, (**b**) C1s, and (**c**) Cu2p3.

The XPS analysis was done to study the elements present, including possible impurities in the Cu, GN, and GN@CR-Cu coatings. In the case of pure Cu, peaks corresponding to C1s, O1s, and Cu2p3 are observed (Figure S2). The presence of a small percentage of O1s is due to the slight oxidation of Cu upon photothermal treatment. In the case of GN, peaks corresponding to C1s and O1s are present. The O1s depict small functionalization or a slightly unreduced part of GO. The survey scan of GN@CR-Cu showed the peaks corresponding to C1s, O1s, and Cu2p3, but here, in contrast to the spectra of pure Cu, a high percentage of C1s and Cu2p3 was observed (Figure 5). The C1s peak can be deconvoluted into three peaks at 284.8, 286.1, and 288.3 eV, corresponding to the sp2 and sp3 (C=C/C–C) bonding, C-O chemical bonds, and carbonyl groups (C=O/COO) in rGO [34,35,36]. The Cu2p peak that can be deconvoluted into the two peaks at 935.1 and 934.8 eV were attributed to the Cu^2+^ and Cu^+^ chemical states [37]. The peak at 942.5 eV is due to the shakeup satellite peak [38].

### 2.3. Antibacterial Performance of GN@CR-Cu Composite

**Figure 6:**
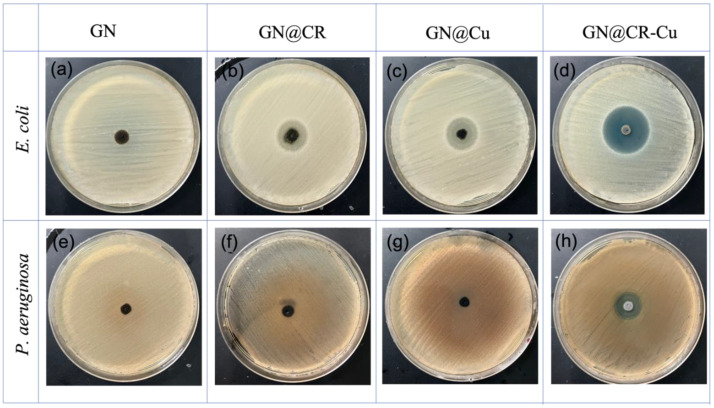
Test bacteria *E. coli* (**a**–**d**), *P. aeruginosa* (**e**–**h**): The zone inhibition by the nanocomposite material on the bacteria cultivated on nutrient agar media plates.

In this study, the antimicrobial testing of GN, GN@CR, GN@Cu, and GN@CR-Cu was conducted against *E. coli* and *P. aeruginosa* bacterial strains. Among these, the GN@CR-Cu composite material showed highly efficient antimicrobial activities against both nosocomial bacterial strains. The *E. coli* bacterial strain was more significantly affected as compared to the *P. aeruginosa* bacterial strain in both assays. The nanocomposite can act by bypassing drug resistance mechanisms in bacteria and inhibiting biofilm formation or other important processes related to their virulence potential [39]. Nanoparticles can penetrate the cell wall and membrane of the bacteria and disrupt important molecular mechanisms [40]. In general, *E. coli* is a facultative anaerobic bacterial species, while *P. aeruginosa* is an aerobic bacterial species. *P. aeruginosa* promoting *E. coli* biofilm formation in a nutrient-limited medium has been reported earlier [41]. *P. aeruginosa* can produce more exopolysaccharides than *E. coli*. These *P. aeruginosa* exopolysaccharides play an important role in biofilm formation, and due to it, *P. aeruginosa* rapidly forms biofilm compared to *E. coli*. Therefore, this exopolysaccharide inhibits the binding of nanomaterial of the *P. aeruginosa* bacterial cell membrane and is hypothesized to contribute to being less affected when compared to *E. coli*. The bacterial strain of *E. coli* and *P. aeruginosa* growth was significantly influenced and developed a bacterial growth inhibition zone around the nanocomposite material, where the compound diffused into the surrounding media. The GN@CR-Cu showed a significant zone of inhibition of 24 ± 0.50 and 18 ± 0.25 mm against *E. coli* and *P. aeruginosa* at 25 µg/mL, while GN@Cu showed 18 ± 0.25 and 14 ± 0.5 mm against *E. coli* and *P. aeruginosa* at a 25 µg/mL concentration, and GN@CR showed 17 ± 0.75 and 12 ± 0.5 mm zone inhibition against *E. coli* and *P. aeruginosa* at the 25 µg/mL concentrations, respectively (Figure 6).

**Figure 7:**
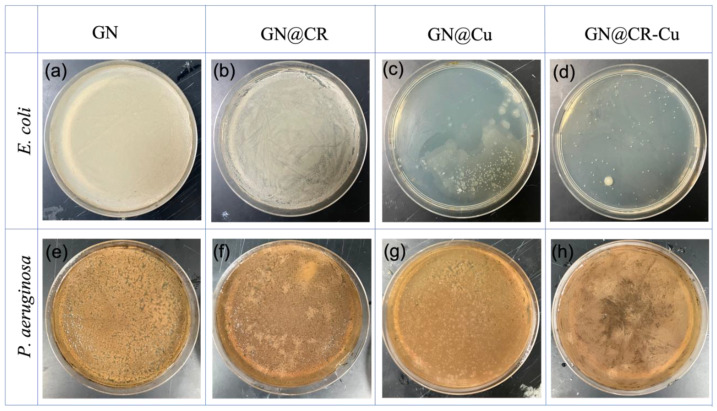
Test bacteria *E. coli* (**a**–**d**), *P. aeruginosa* (**e**–**h**): The minimum inhibition by the nanocomposite material on the bacteria cultivated Petri plates.

**Figure 8:**
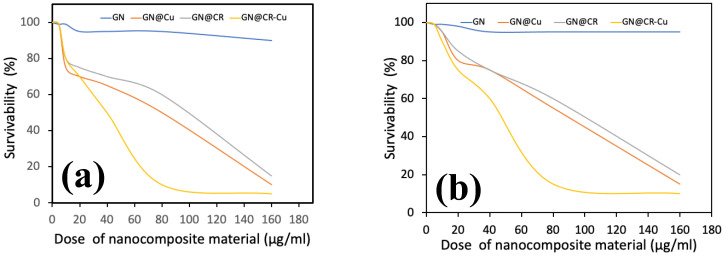
The survivability of *E. coli* (**a**) and *P. aeruginosa* (**b**) in the presence of nanocomposite material. Image showing excellent dose-response, increasing concentration significantly retards bacterial growth.

In addition, antibacterial activity was examined by testing it in a broth and determining its minimum inhibitory concentrations (MIC) and minimum bactericidal concentrations (MBC). It was reported that the inhibitory concentration against both microorganisms individually may reach up to 80, 160, and 160 µg/mL for GN@CR-Cu, GN@CR, and GN@Cu, respectively. The antibacterial activity of the composites was evaluated by putting it into a liquid culture nutrient broth along with the inoculated test bacteria and subsequently inoculating it as described in Section 3.2.1. After overnight incubation, a clear pattern of bacterial growth was found on the nutrient agar plate, which is shown in Figure 7. Further antimicrobial work was performed in terms of bacterial survivability in the presence of composite materials (Figure 8). GN alone did not significantly inhibit bacterial growth in the cases of both *E. coli* and *P. aeruginosa*. The GN composites with either CR or Cu were effective antibacterial agents; however, their activity was less potent when compared to the GN@CR-Cu composite (Figure 8a,b).

We hypothesize that the GN@CR-Cu antibacterial mechanism combines both chemical and physical modes of action. GN enriched with the chemical agent Cu and biological or natural antimicrobial agent CR showed superior antibacterial activity when compared to other formulations (i.e., GN, GN@CR, or GN@Cu). We hypothesize that when the nanocomposite material encounters bacterial cells, the sharp edge of the GN pierce the bacterial membranes causing the leakage of cell organelles. Similarly, when the nanocomposite is coated on solid surfaces, the water contact angle measured ~70° (Figure S3). We hypothesize that the hydrophilic surface might contribute to the decreased bacterial attachment ability of the strains tested [42,43]. It might also be possible that the creation of various active oxygen species occurs, which prevents the growth of bacterial cells due to the presence of Cu in the formulation [44]. In the previously published studies, it was demonstrated that Cu NPs have outstanding antibacterial activity. In a recent study, the Cu nanoparticle size and concentration have a direct effect on the antimicrobial activity of *E. coli* via numerous mechanisms [45].

**Figure 9:**
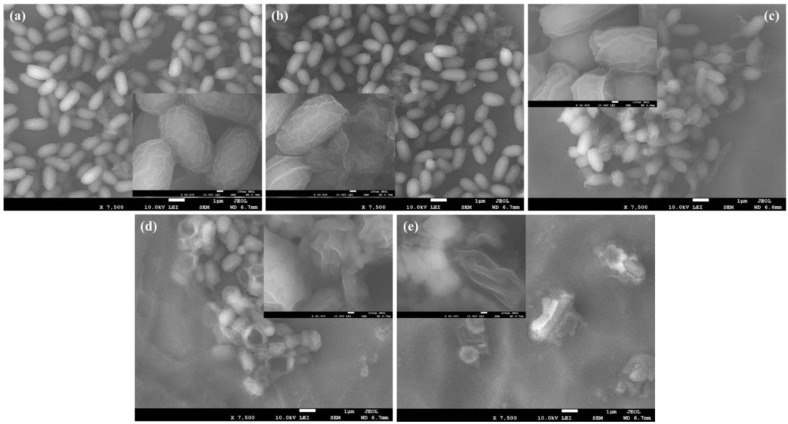
Scanning electron microscopy image of *E. coli* without any treatment as a control without an effect on cell morphology (**a**), and *E. coli* culture treated with GN (**b**), GN@CR (**c**), partial cell damage by the treatment of GN@Cu (**d**), and complete bacterial cell damage by the treatment of GN@CR-Cu (**e**).

In this study, GN deposited with Cu nanomaterial significantly enhances its antimicrobial activity clearly shown in the zone inhibition (Figure 6, Figure 7 and Figure 8) and electron microscopy image (Figure 9) studies. This material was further enriched with the addition of natural CR, which reveals excellent antimicrobial activity. Recently, the antibacterial potential of bulk CR and nano CR against the *Staphylococcus aureus* and *E. coli* was successfully investigated by Sandhuli et al. [46]. The inhibition zones of the nano-formulated CR cream were greater than those of bulk CR cream for both *S. aureus* and *E. coli*, demonstrating its superior antibacterial action [46]. In our case, the GN@CR-Cu showed better antimicrobial potential than GN@Cu and GN@CR alone, most likely due to the synergistic effect of the two antimicrobial agents [47].

The antibacterial mechanisms involved in the bactericidal activity of GN-containing nanomaterials, in general, could be summarized using the following mechanisms: (i) physical direct interaction of the extremely sharp edges of nanomaterials against the cell wall membrane [48], which can show further stress under sonication, (ii) ROS generation [49] even in the dark [50], (iii) trapping cells within the aggregated nanomaterials [7], (iv) oxidative stress [51], (v) interruption in the glycolysis process of the bacteria [52], (vi) DNA damage [53], (vii) metal ion release [54], and recently, (viii) contribution in generation/explosion of nanobubbles [55]. In the case of our nanocomposite, we hypothesize that the multiple modes of actions are at play, including bacterial cell damage that occurs due to the nanoparticle interaction. GN@Cu-CR materials are multifunctional due to the metal presence and natural CR. In order to prevent microbes from attaching, colonizing, spreading, and creating biofilms in medical devices, composite materials have the potential for external uses as antibacterial agents in the surface coatings on a variety of substrates.

Here, the nanocomposite material has been synthesized with a highly stable material, i.e., GN which stabilizes both CR and Cu. Due to the high stability of GN-based materials, the CR and Cu molecules fixed in the GN groves retain their antimicrobial activity. In our previous reports, the GN-based nanocomposite material with Zinc oxide and CR showed similarly excellent stability and antimicrobial activity against the multidrug-resistant *Staphylococcus aurous* bacterial strain [33].

## 3. Materials and Methods

### 3.1. Materials

Copper sulphate pentahydrate CuSO_4_·5H_2_O was sourced from Fluka (Buchs, Switzerland), and cetyltrimethylammonium bromide (CTAB) from Otto chemicals (Mumbai, India). Sulphuric acid, phosphoric acid, potassium permanganate, and ascorbic acid (C_6_H_8_O_6_) were obtained from Sigma Aldrich (St. Louis, MO, USA). CR was purchased from a local supermarket of Jeddah, Saudi Arabia, and was dried under the Sun and subsequently crushed into fine powder. The water used in the experiments was deionized water.

#### 3.1.1. Synthesis of Cu and GO@CR-Cu Suspension

The Cu nanoparticles were synthesized by the reduction of copper (II) sulfate in the presence of CTAB surfactant. In a typical process, 0.1 M copper (II) sulfate solution was dissolved in 100 mL of water, and to it, 0.25 g of CTAB was added and the whole system was put under stirring conditions. In another beaker, 50 mL of 0.2 M ascorbic acid solution was prepared. In the second step, the solution of ascorbic acid was slowly added to the copper (II) sulfate solution, and subsequently, 30 mL of 1 M sodium hydroxide solution was also added. The whole system was heated to 80 °C for 2 h and a dark reddish-brown color confirmed the formation of Cu. Thus, the prepared Cu was separated by centrifugation, washed with an excess of water and ethanol, and subsequently dried at room temperature [56].

GO was prepared using the modified Tour’s method. In a typical process, to a 9:1 stirring mixture of concentrated H_2_SO_4_/H_3_PO_4_ (360:40 mL), 18 gms of KMnO_4_ followed by 3 g of graphite flakes was added slowly. The whole system was left stirring at room temperature for 72 h for the exfoliation of graphite flakes. Thereafter, the mixture was cooled by putting it in an ice bath and was then subsequently poured into another beaker containing 400 mL of deionized water ice. To this cooled solution, 1–3 mL 30% H_2_O_2_ was added until the appearance of a yellow color confirmed the formation of GO. Thus, the synthesized GO was separated by centrifugation/filtration, washed to 100 mL of 5% HCl, excess of water, ethanol, and subsequently stored as a gel [57]. The GO solution was optimized and a stock solution of 10 mg/mL was prepared for future use.

#### 3.1.2. Preparation of GO@CR-Cu Slurry

To 50 mL of the GO (10 mg/mL) solution, 0.125 g of both Cu and CR was added. The whole system was put on ultrasonic bath and later kept on stirring for the uniform distribution of Cu and CR in the GO. Just before coating, the mixture was repeatedly stirred/shaken for the uniform distribution of Cu and CR. For the reduction of GO inside GO@CR-Cu, the coated GO@CR-Cu was kept in sunlight on a bright sunny day for 8 h from 8 a.m. to 4 p.m. in Jeddah. For the evaluation of antimicrobial activity, the above GO@CR-Cu composite was taken in a Petri plate, reduced into GN@CR-Cu by the process described earlier, and finally used in its powdered form.

#### 3.1.3. Characterization

The morphological and compositional analysis of GN@CR-Cu were conducted by field emission scanning electron microscopy (FESEM) (JSM-7600F from JEOL, Tokyo, Japan). The elemental mapping was recorded using the energy dispersive X-ray spectroscope (EDS) from Oxford Instruments, Oxfordshire, UK equipped with FESEM. For the elemental detection, X-ray photoelectron spectroscopy (XPS) (ESCALAB 250 from Thermo Fisher Scientific, Warrington, UK) was used at a monochromatized Al Kα X-ray source λ 1/4 1486.6 eV. The antibacterial studies were performed by studying the surface growth inhibition assay, and the minimum inhibitory concentration (MIC) and minimum bactericidal concentration (MBC) were determined against the *Escherichia coli* and *Pseudomonas aeruginosa* microorganisms.

### 3.2. Assessment of Antibacterial Activity of GN@CR-Cu Composite

#### 3.2.1. Zone Inhibition Assay

The two bacterial strains, *Escherichia coli*, and *Pseudomonas aeruginosa* are frequently linked to nosocomial infections. This experiment investigated the antibacterial capability of materials that were synthesized. To obtain the best growth acquisition, these bacterial cultures were first grown in a Luria Bertani broth, and then a loopful culture was injected into the 100 mL liquid broth and incubated in a rotatory incubator. A fresh culture of each strain was created by re-culturing with the same medium under the ideal circumstances. The nutrition agar plate was created by utilizing the pour plate technique after the medium was autoclaved for 20 min in a sterilizer machine at the proper temperature of 121 °C and pressure of 15 pounds per square inch (JSR Autoclave, JSAC-80, Gongju-Cit, Republic of Korea). The media was poured into the Petri plates and solidified after 10 min of sitting. Each bacterial strain was moved from the fresh culture tubes to the media plates and then left there for 10 min to maintain the conditions the medium on the plate afforded. A total of 25 micrograms of the synthesized composite materials were added to the surface of each media plate, which previously contained bacteria. The nanocomposite material came into contact with the bacteria and spread into the surrounding media after an overnight incubation at the ideal temperature of 37 °C, revealing the antibacterial activity around the composite materials and developing a clear zone of bacterial growth inhibition.

#### 3.2.2. MIC and MBC of GN@CR-Cu Composite

The effect of GN@CR-Cu nanocomposite on the growth of bacteria in broth was investigated, and the lowest concentrations required to impede growth (MIC) and kill bacteria (MBC) were determined. The GN@CR-Cu nanocomposite was used as an antibacterial agent against specific test microorganisms after being suspended in Milli-Q water. The broth dilution process is the approach that is the industry standard for assessing bacterial survival in the presence of the tested test agent or nanocomposite material. The concentration was adjusted by adding nanomaterials from 0 to 100 µg/mL. In the beginning, the same techniques were followed to set up twelve 500 mL flasks, each of which contained 100 mL of broth media. These flasks were then sterilized in an autoclave at standard conditions. The McFarland 0.5 methodology was used to standardize the bacterial culture and examine the compounds’ antibacterial susceptibility. In addition, the medium was supplemented with composite materials from 25 to 100 µg/mL before being grown in a rotatory incubator at 37 °C for an incubation period of 16 h. Using a broth medium, the nanocomposite materials were serially diluted before being injected with a bacterial culture containing 5 × 10^6^ CFU for a 16 h incubation period. Bacterial culture turbidity and plate count were assessed following the incubation period.

#### 3.2.3. Bacterial Survivability with Nanomaterials

In this study, the spot-plating method was used to quantify bacterial growth. The assay utilizes the principle that a bacterial culture would have a decreasing percentage of viable cells with increasing concentrations of anti-microbial compounds. At a constant sub-MIC level of an agent, the sensitivity of a bacterial strain could be correlated with the percentage of cells that survive and form a colony. This ratio of surviving cells to the total number of plated cells is the plating efficiency. The key to a successful spot-plating assay is to spot-plate the same number of cells onto each spot. By comparing the surviving cells on the material containing plates to the control, the sensitivity of the cells to the nanocomposite concentration by plating efficiency percentage can be determined.

*Determination of the optimal sub-MIC level of nanomaterials*: The chosen sub-MIC level nanomaterials should be chosen with care. The concentration should be high enough to inhibit growth, but not to inhibit all growth. This optimal sub-MIC concentration is usually just below the MIC of the nanomaterials. For example, the MIC of nanomaterials and *E. coli* is 50 μg/mL. The optimal sub-MIC of nanomaterials for the spot plate assay was found to be 40 μg/mL. Media preparation for bacterial growth with and without nanomaterials should be made. The bacterial stocks should be grown overnight at their optimal growth conditions and maintained according to the McFarland standard, culture optimum incubation at 37 °C, and shaking at 250 rpm. After overnight growth, the culture should be diluted to a concentration that would allow countable isolated colonies on the final spots of the LB plates. We recommend dilutions of 1 × 10^4^ CFU/mL. A small volume (10 μL) of the working stock of bacterial culture should be plated up to six times on each plate. The number of colonies in each spot should be counted and tallied. The number of colonies on the antibiotic plates and the control plates are used to calculate.

*Nanomaterials stock preparation*: The amount of the required nanomaterial concentration was weighed by analytical balance and then transferred and dissolved with the required amount of sterile dH2O, before being filtered through a 0.20 μm filter into a sterile 10 mL falcon tube.

*Dilution of bacterial culture to a working concentration:* The bacterial culture turbidity and availability of bacteria count was determined by spectrophotometer at OD600. The obtained value was multiplying the OD600 of the bacterial culture by the appropriate conversion ratio of OD600 to CFU/mL (if OD600 = 8 × 10^8^ CFU/mL). Further specific required dilution of the working concentration was also determined.

*Spot plating*: First of all, the media plates were prepared and supplemented with and without nanomaterials of interest at the desired concentrations of 0 to 100 µg. The culture tubes were inoculated and incubated overnight at 37 °C. The next day, the optical density of the diluted cultures was determined and converted into CFU/mL using the conversion factor of the strain, if known (1 OD at 600 equals to 8 × 10^8^ cells/mL). Further, the cultures (100 μL of culture into 900 μL of fresh growth media) were serially diluted to obtain a 1 × 10^4^ CFU/mL culture. An aseptic pipette was used to separate the spots onto a plate using 10 μL of 1 × 10^4^ CFU/mL culture. After incubation, the isolated colonies per spot were counted. The number of colonies on each spot of the nanomaterial’s plates were also counted.

### 3.3. Imaging of Bacterial Growth Inhibition by SEM

In this study, both test bacterial strain fresh cultures were taken according to the McFarland standard and centrifuged and treated with the nanocomposite material, according to the MIC concentration, for 16 h incubation at 37 °C before being centrifuged at 8000 rpm for 10 min. The bacterial pellets were selected and washed multiple times with phosphate saline buffer and treated with 2% glutaraldehyde solution and the bacterial sample was placed at 4 °C for proper fixing. After fixing, the bacterial sample was washed with double distilled water, then further washed with 10 to 100 % ethanol in increasing order, and the obtained bacterial culture was then mounted on the stab of SEM and analyzed.

## 4. Conclusions

In this study, the nanocomposite material GN@CR-Cu was successfully synthesized and coated on contact surfaces for studying its antimicrobial activity. The antimicrobial coatings of GN@CR-Cu were prepared using the chemical synthesis methodology and were further characterized using electron microscopy and X-ray photon spectroscopy. GN@CR-Cu showed excellent antimicrobial effects against *E. coli* and *P aeruginosa* bacterial isolates. The nanocomposite showed antimicrobial activity, most likely by contact-killing mechanisms, which was suggested by zone inhibition and scanning electron microscopy. The materials showed maximum antibacterial activity against *E. coli* (24 ± 0.50 mm) followed by *P. aeruginosa* (18 ± 0.25 mm) at 25 µg/mL spot inoculation on the solid media plate, and a similar trend was observed in the minimum inhibition concentration (80 µg/mL) and bactericidal concentration (160 µg/mL) in liquid media. According to this proof-of-concept study, GN@CR-Cu can function as a potent future anti-microbial nanomaterial for the prevention of nosocomial infection, if coated on medical devices or food preparation instruments.

## Data Availability

The data presented in this study are available on request from the corresponding author.

## Supplementary Materials

The following supporting information can be downloaded at: https://www.mdpi.com/article/10.3390/molecules28062814/s1, Figure S1: FESEM image of (a) CR and (b) Cu nanoparticles, Figure S2: XPS survey scan of GN and Cu, Figure S3: Water contact angle measurement of GN@CR-Cu composite.
