# Supplementary material for: Graphene@Curcumin-Copper Paintable Coatings for the Prevention of Nosocomial Microbial Infection
Source: Molecules. 2023 Mar 20;28(6):2814. doi: 10.3390/molecules28062814 (PMC10051306; doi:10.3390/molecules28062814)
Supplement: Supplementary file 1 [file molecules-28-02814-s001.zip › molecules-2237675-supplementary.pdf]

molecules

MDPI

Article

Graphene@Curcumin-Copper Paintable Coatings for the Prevention of Nosocomial Microbial Infection

Mohammad Oves [1], Mohammad Omaish Ansari [2], Mohammad Shahnawaze Ansari [2], and Adnan Memić [2,*]

[1] Center of Excellence in Environmental Studies, King Abdulaziz University, Jeddah 21589, Saudi Arabia
[2] Center of Nanotechnology, King Abdulaziz University, Jeddah 21589, Saudi Arabia
* Correspondence: amemic@kau.edu.sa; Tel.: +966-564975404

Citation: Oves, M.; Ansari, M.O.; Ansari, M.S.; Memić, A. Graphene @curcumin-copper paintable coatings for the prevention of nosocomial microbial infection. *Molecules* **2023**, *volume number*, x. https://doi.org/10.3390/xxxxx

Academic Editor(s):

Received: date
Revised: date
Accepted: date
Published: date

Publisher's Note: MDPI stays neutral with regard to jurisdictional claims in published maps and institutional affiliations.

Copyright: © 2023 by the authors. Submitted for possible open access publication under the terms and conditions of the Creative Commons Attribution (CC BY) license (https://creativecommons.org/licenses/by/4.0/).

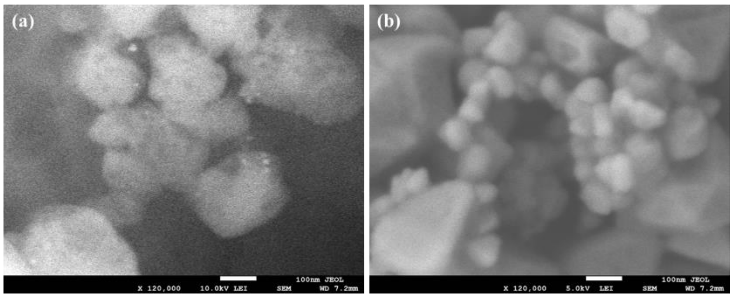

Figure S1. FESEM image of (a) CR and (b) Cu nanoparticles. Figure S1(a-b) shows high resolution FESEM images of CR and Cu. It is clearly evident from Fig. S1(a) that the CR nanoparticles have fluffy structure with critical size nanopores on its sufaces. On the other hand, Cu nanoparticles have solid gemetry showing polyhedral shapes [Fig. S1(b)]. Also, it seems that both the samples are highly uniform, dispersed and well seperated with any lump or aglomeration.

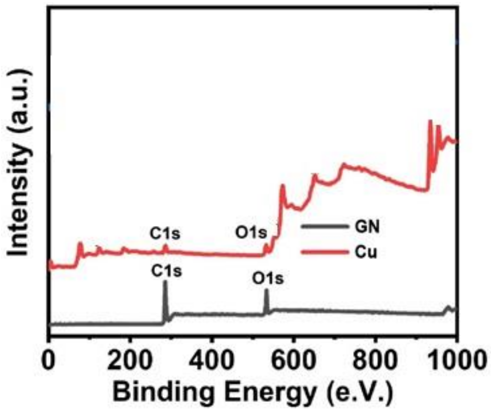

Figure S2. XPS survey scan of GN and Cu.

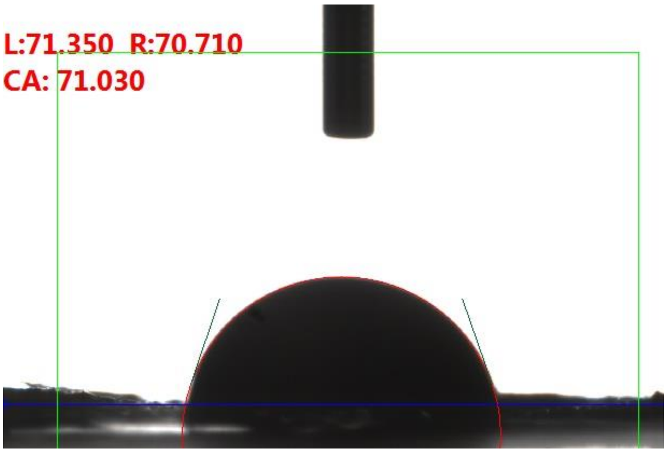

Figure S3. Water contact angle measurement of GN@CR-Cu composite.
